# A multi-center study on the risk factors of infection caused by multi-drug resistant *Acinetobacter baumannii*

**DOI:** 10.1186/s12879-017-2932-5

**Published:** 2018-01-05

**Authors:** Huiping Huang, Borong Chen, Gang Liu, Jing Ran, Xianyu Lian, Xinhua Huang, Nan Wang, Zhengjie Huang

**Affiliations:** 1 Department of Infection Control, The First Affiliated Hospital of Xiamen University, Xiamen, Fujian 361003 China; 2 Department of Gastrointestinal Surgery, Xiamen Cancer Hospital, The First Affiliated Hospital of Xiamen University, 55 Zhen Hai Road, Si Ming District, Xiamen, Fujian 361003 China; 3 Department of Gynecology and Obstetrics, The First Affiliated Hospital of Xiamen University, Xiamen, Fujian 361003 China; 4 Department of Infection Control, The Second Hospital of Xiamen, Xiamen, Fujian 361021 China; 5 Department of Infection Control, The Affiliated Chenggong Hospital of Xiamen University, Xiamen, Fujian 361003 China

**Keywords:** Intensive care unit, Multi-drug resistant, *Acinetobacter baummanii*, Risk factors

## Abstract

**Background:**

*Acinetobacter baumannii* (AB) is critical for healthcare-associated infections (HAI) with significant regional differences in the resistance rate, but its risk factors and infection trends has not been well studied. We aimed to explore the risk factors, epidemiological characteristics and resistance of multidrug-resistant *Acinetobacter baumannii* (MDR-AB) in intensive care unit inpatients.

**Methods:**

Data of patients with MDR-AB (195 cases), and with antibiotic-sensitive AB infection (294 cases, control) during January to December, 2015 in three medical centers in Xiamen, China were conducted and analyzed in the present retrospective study.

**Results:**

Lower respiratory tract infection with AB accounted for 68.71%. MDR-AB was detected in 39.88% of all cases. Univariate analysis suggested that mechanical ventilation, indwelling catheter, cancer patients, length of hospitalization in intensive care unit (ICU) ≥15 d, Acute Physiology and Chronic Health Evaluation (APACHE) II score, combined using antibiotic before isolation of AB and use of third-lines cephalosporins were associated with the development of MDR-AB healthcare-associated infections. Dose-response relationship analysis suggested that the age and the days of mechanical ventilation were associated with increased infection with MDR-AB. Logistic regression analysis suggested that, mechanical ventilation, combined using antibiotic before isolation of AB, and indwelling catheter, were associated with MDR-AB infection, with odds ratios (OR) and 95% confidence intervals (CI) of 3.93 (1.52–10.14), 4.11 (1.58–10.73), and 4.15 (1.32–12.99), respectively.

**Conclusions:**

MDR-AB infection was associated with mechanical ventilation, combined using antibiotic before isolation of AB, and indwelling catheter. Furthermore, the age and the days of mechanical ventilation were associated with increased infection with MDR-AB.

## Background

Acinetobacter baumannii (AB) is a Gram-negative, lactose non-fermenting organism and its ability to survive in hospital environments, which is increasingly becoming a major healthcare-associated infections (HAI) pathogen worldwide. With the emergence of HAI, AB is an important cause in critically ill patients. AB is now largely regarded as one of the most troublesome pathogens and is responsible for several types of HAI including skin and soft tissue infections and invasive infections, such as pneumonia, osteomyelitis, and bacteremia [1].

Reports about multi-drug resistance *Acinetobacter baumannii* (MDR-AB) has been constantly increased, especially in the intensive care units (ICU) [2–4]. MDR-AB-caused infections are difficult to diagnose and treat, leading to increased mortality and prolonged hospital stays [5]. A recent study demonstrated that the 30-day hospital mortality rate of bloodstream infections caused by MDR-AB was 55.2% in geriatric inpatients [1].

Carbapenem resistant rate increases every year. It was showed that some hospital ICU emerged carbapenem resistant (CR) AB. Resistance of AB isolates to imipenem dramatically increased from16% in 2003–2007 to 78% in 2008–2010 in a single tertiary hospital in South Korea [6, 7]. Cross-transmission of MDR-AB was common in the hospital, resulting in ICU and neonatal ward infection outbreaks and epidemics, and was correlated with an adverse outcome, including an independent predictor of death and complications [8–11].

A large amount of data is available regarding the epidemiology, risk factors, and outcomes of patients with AB. However, besides the rapid growth worldwide, there are significant regional differences in the resistance rate of AB. Information is limited regarding the risk factors for MDR-AB in ICU inpatients in developing countries.

Therefore, we designed the present retrospective study to investigate the risk factors and infection trends caused by MDR-AB in ICU inpatients. Patients in three university affiliated hospitals with more than 800 beds in Xiamen, China, were evaluated. The clinical characteristics as well as the trends of MDR-AB with the dose-response relationship were analyzed. Our study is beneficial for understanding clinical significance and risk factors of MDR-AB, and for providing support for future management in ICU clinical practice.

## Methods

### Setting

Three ICU of tertiary general hospitals in Xiamen, including the First Affiliated Hospital of Xiamen University, the No. 2 Hospital of Xiamen, and 174th Hospital of the Chinese People’s Liberation Army (the Affiliated Chenggong Hospital of Xiamen University), of which the microbial identification results are homogeneous and are regulatory accepted by each other, were selected by using stratified sampling method. Four hundred eighty-nine patients who were hospitalized during the period of January 1st to December 31st, 2015 patients with AB infection were included as participants in the present study. Repeated strains isolated from the same patient in the same part of the specimen were excluded.

The quality control methods of sputum samples: Under laboratory microscope, if the squamous epithelial cells in the low fold field of vision <10 and the white blood cells >25 are qualified samples.

Diagnostic criteria: The designation of MDR was defined as the absence of susceptibility to >3 of the following antimicrobials or groups of antimicrobials: ampicillin/sulbactam, aztreonam, ceftazidime, ciprofloxacin, gentamicin, imipenem, piperacillin, trimethoprim/sulfamethoxazole, carbapenems, and amikacin [12, 13].Bacterial isolation and antimicrobial susceptibility testing were performed in accordance with the methodology of the Clinical and Laboratory Standards Institute [14]. HAI was confirmed according to the surveillance definition of the Centers for Disease Control and Prevention/National Healthcare Safety [15].

### Investigation methods

The cases were reviewed in the microbiology and inspection laboratory database by searching for at least 2 positive cultures. Through a retrospective survey, the medical records of the cases were obtained from the medical record archives. The demographics, information, regarding clinical care, microbiologic data, treatments provided, and outcomes of the patients with clinically significant AB were reviewed.

Main contents of questionnaire including the name, gender, age, APACHE II score, comorbidities, ICU days, infection site, HAI, hospitalization days before infection, operation, invasive operation, antibiotic use, and drug sensitivity were collected. The data were analyzed after double-entry.

### Definitions of main risk factors

1) Comorbidities refer to hypertension, coronary heart disease, diabetes, cancer, chronic renal insufficiency, cerebral infarction, etc. 2) ICU days refers to patients with ICU admission until test positive for the first time. 3) The combination of antimicrobial agents is the use of 2 or more than 2 kinds of antibacterial drugs. 4) Invasive operation refers to tracheotomy, nasal feeding, indwelling catheter, arteriovenous catheter, abdominal puncture, ventilator etc.

### Statistical analysis

All of the statistical analyses were performed using SPSS 13.0 (SPSS Inc., Chicago, IL, USA). Categorical variables were analyzed using the Χ^2^ test or Fisher’s exact test, and continuous variables were analyzed using Student’s t test or Mann-Whitney U test, and were generally presented as means and standard deviation. Multivariate logistic regression analyses using the forward likelihood ratio selection method were used. To identify independent factors of MDR-AB and is presented with an odds ratio (95% confidence intervals, CI). Potential candidate variables were those with *P* < 0.05 in univariate analyses. All of the *P* values were 2 tailed, and *P* < 0.05 was considered statistically significant.

## Results

### Clinical characteristics

A total of 489 patients aged from 38 days to 101 years with AB isolations during January to December 2015 were identified in this study. The mean age of the patients was 56.54 ± 27.18 years. The male to female ratio was 3.9:1(Male 390, female 99). Two hundred twenty-one of the cases were healthcare-associated infections, accounting for 45.20%.

MDR-AB was identified in up to 195 cases (39.88%), male 165 and female 30. Two hundred ninety-four cases were Non-MDR-AB, male 225 and female 69. The gender was significantly associated with the infection caused by MDR-AB (Χ^2^ = 4.74, *P* = 0.03).

The average age of the patients (64.93 ± 21.13) was significantly higher than that (50.98 ± 29.28) in the control group (*t* = 5.74, *P* < 0.001).

The positive AB isolations were common in the respiratory tract (72.31% versus 66.33%, respectively). There was no significant difference between the two groups (Table 1).

**Table 1:** Source of *Acinetobacter baummanii* from clinical specimens

Group	Sputum	Whole blood	Drainage fluid	Central venous catheter tip	Others
	N%	N%	N%	N%	N%
MDR-AB	141 (72.3)	9 (4.6)	21 (10.8)	10 (5.1)	14 (7.2)
Non-MDR-AB	195 (66.3)	23 (7.8)	29 (9.9)	12 (4.1)	35 (11.9)
Total	336 (68.7)	32 (6.5)	50 (10.2)	22 (4.5)	49 (10.0)

Χ^2^ = 5.45, *P* = 0.24

### Univariate analysis for risk factors of MDR-AB

The Χ^2^ analysis results show, HAI, mechanical ventilation, indwelling catheters, ICU days, APACHE II score, combined use of antimicrobial agents prior to infection and the use of third-generation cephalosporins, hospitalization ≥3 times, history of cancer are associated with MDR-AB infection. And the operation history, comorbidities and other factors are no significant correlated with MDR-AB infection (Table 2).

**Table 2:** The results of one-way Chi-square test about exposure factors in MDR-AB infection patients

Characteristics		*Acinetobacter baumannii* bacteremia N (%)		*P*
		MDR-AB (*N* = 195)	Non-MDRAB (*N* = 294)	
Sex	Male	165 (84.6)	225 (76.5)	0.03
	Female	30 (15.4)	69 (23.5)	
Age (y)	< 50	10 (5.13)	58 (19.7)	
	50–60	22 (11.3)	131 (44.6)	< 0.001
	60–70	98 (50.3)	62 (21.1)	
	70–80	46 (23.6)	34 (11.6)	
	> 80	19 (9.74)	9 (3.06)	
Recent history of surgery	Yes	54 (27.7)	75 (25.5)	0.59
	No	141 (72.3)	219 (74.5)	
Comorbidities	< 2	165 (84.6)	246 (83.7)	0.78
	≥2	30 (15.4)	48 (16.3)	
HAI	Yes	117 (60.0)	104 (35.4)	< 0.001
	No	78 (40.0)	190 (64.6)	
ICU days	< 15d	24 (12.3)	78 (26.5)	< 0.001
	≥15d	171 (87.7)	216 (73.5)	
Mechanical ventilation	Yes	177 (90.8)	210 (71.4)	< 0.001
	No	18 (9.2)	84 (28.6)	
Indwelling catheters	Yes	183 (93.8)	234 (79.6)	< 0.001
	No	12 (6.2)	60 (20.4)	
Combined use of antimicrobial agents prior to infection	Yes	174 (89.2)	246 (83.7)	< 0.001
	No	21 (10.8)	48 (16.3)	
Use of third-generation cephaloglycin	Yes	186 (95.4)	246 (83.7)	< 0.001
	No	9 (4.6)	48 (16.3)	
Hospitalization times	< 3	33 (16.9)	97 (33.0)	< 0.001
	≥3	162 (83.1)	197 (67.0)	
History of cancer	Yes	121 (62.1)	148 (50.3)	0.01
	No	74 (37.9)	146 (49.7)	
APACHE II score	< 18	117 (60.0)	228 (77.6)	< 0.001
	≥18	78 (40.0)	66 (22.4)	

### Multivariate regression analysis results

An unconditional logistic multiple regression analysis was performed on 9 variables with significant differences in single factor analysis. Finally logistic multiple regression analysis revealed that the mechanical ventilation, combined use of antibacterial drugs before infection, and indwelling catheter were independent risk factors associated with the MDR-AB infection, respectively (Table 3).

**Table 3:** The results of unconditional logistic multiple regression analysis about the risk factors of MDR-AB infection

Risk factors	B (k)	SE (B)	Χ^2^	*P*	*OR*	95% *CI*
Mechanical ventilation	1.37	0.48	8.03	< 0.001	3.93	1.53–10.14
Combined use of Antimicrobial agents	1.41	0.49	8.35	< 0.001	4.11	1.58–10.73
Indwelling catheter	1.42	0.58	5.97	0.02	4.15	1.33–13.00

### Dose-response relationship

The trend Χ^2^ analysis showed that there was a dose-response relationship between the age, the days of mechanical ventilation, and MDR-AB infections, respectively. The age and the days of mechanical ventilation are both positively associated with the MDR-AB infections (Table 4).

**Table 4:** The results of tendency chi-square test about the relation of MDR-AB infection with the age and the days of mechanical ventilation

Exposure factors	Observed cases	Cases of MDR-AB	Infection rate (%)	OR	Χ^2^	*P*
Age (years)						
0	13	2	15.38	1.00	58.62	< 0.001
20	69	16	23.19	1.36		
40	127	37	29.13	1.41		
60	184	72	39.13	1.64		
80	96	68	70.83	3.43		
Mechanical ventilation (days)						
0	102	18	17.65	1.00	43.36	< 0.001
10	247	94	38.06	2.87		
20	140	83	59.29	6.80		

## Discussion

MDR-AB infection could significantly prolong the hospital stay, increase mortality, and increase economic costs [8, 11]. The present study was a retrospective, observational, multi-center study. Multiple regression analysis was employed to minimize the confounding bias. We showed that the incidence of MDR-AB was 39.88% in ICU inpatients, and was less than 56.3%, while more than 50% of the positive AB specimens were from sputum and lower respiratory tract infection was predominant. Our results were in concert with the studies reported by Zorgan, Dejsirilert, Custovic, etc. [2, 16–19]. In addition, we analyzed the relationship between the use of antibacterial agents and MDR-AB infection. It is found that combined use of antibiotics before the infection is an important factor affecting the incidence of MDR-AB.

Studies had shown that tumor history, high APACHE II score and indwelling catheter were risk factors with the infection of resistant bacteria [9, 20, 21]. By using univariate analysis, we also found that the risk of MDR-AB infection in the patients with hospitalization ≥3 times was 2.42 fold of that in patients with hospitalization <3 times.

In our study, a significant association between MDR-AB infection and mechanical ventilation, combined use of antimicrobial agents, indwelling catheters were noted. There were independent risk factors for MDR-AB in ICU inpatients. Mechanical ventilation is often considered as an important factor in the MDR Acinetobacter pneumonia [4, 22, 23]. In the present study, mechanical ventilation was in 187 of 195 cases (accounting for 95.89%), whereas lower respiratory tract infections accounted for 72.31%, further confirmed mechanical ventilation as an important factor for MDR-AB infection.

The use of multiple types of antibiotics also increased MDR-AB infections (OR = 4.111), since we also found that combination with antibiotics before infection is an important risk factor. Studies have reported that, use of carbapenem antibiotics within 28 days of infection was significantly related with MDR bacterial infection [21, 23, 24]. Inappropriate drug combination leads to selective pressure, which increases the opportunity of AB infection and promotes the selection of drug-resistant bacteria. The β- lactam antimicrobial drugs could induce AB to produce β-lactamase, and hence inactivate antimicrobial drugs, resulting in resistance of AB to other β-lactam antibiotics [25]. Therefore, the principle of the combination must be strictly followed, and the frequent replacement of antibiotics should be avoided.

Indwelling catheter is also an independent risk factor for MDR-AB infection (OR = 4.149). Similar results were also reported previously [21, 22]. MDR-AB could spread through catheter and puncture point, and treatment device.

The trend Χ^2^ analysis showed that there was a significant dose-response relationship between age, days of mechanical ventilation, and MDR-AB infections. The risk of MDR-AB infections increased with higher age, and with longer time of mechanical ventilation.

## Conclusions

In conclusion, MDR-AB infection was associated with mechanical ventilation, combined using antibiotic before isolation of AB, and indwelling catheter. Furthermore, the age and the days of mechanical ventilation were associated with increased infection with MDR-AB.

## Abbreviations

AB: *Acinetobacter baumannii*
APACHE: Acute Physiology and Chronic Health Evaluation
CI: Confidence intervals
CR: Carbapenem resistant
HAI: Healthcare-associated infections
ICU: Intensive care unit
MDR-AB: Multidrug-resistant *Acinetobacter baumannii*
OR: Odds ratios
